# Climate change and the performance of larval coral reef fishes: the interaction between temperature and food availability

**DOI:** 10.1093/conphys/cot024

**Published:** 2013-10-01

**Authors:** Ian M. McLeod, Jodie L. Rummer, Timothy D. Clark, Geoffrey P. Jones, Mark I. McCormick, Amelia S. Wenger, Philip L. Munday

**Affiliations:** 1 AIMS@JCU and Australian Institute of Marine Science, Townsville, QLD 4810, Australia; 2 School of Marine and Tropical Biology, James Cook University, Townsville, QLD 4811, Australia; 3 ARC Centre of Excellence for Coral Reef Studies, James Cook University, Townsville, QLD 4811, Australia; 4 CSIRO Marine and Atmospheric Research, Brisbane, QLD 4000, Australia

**Keywords:** Connectivity, developmental rate, population viability, thermal reaction norm

## Abstract

Climate-change models predict that tropical ocean temperatures will increase by 2–3°C this century and affect plankton communities that are food for marine fish larvae. Both temperature and food supply can influence development time, growth, and metabolism of marine fishes, particularly during larval stages. However, little is known of the relative importance and potential interacting effects of ocean warming and changes to food supply on the performance of larval fishes. We tested this for larvae of the coral reef anemonefish, *Amphiprion percula,* in an orthogonal experiment comprising three temperatures and three feeding schedules. Temperatures were chosen to represent present-day summer averages (29.2°C) and end-of-century climate change projections of +1.5°C (30.7°C) and +3°C (32.2°C). Feeding schedules were chosen to represent a reduction in access to food (fed daily, every 2 days, or every 3 days). Overall, larvae took longer to settle at higher temperatures and with less frequent feeding, and there was a significant interaction between these factors. Time to metamorphosis was fastest in the 30.7^o^C and high food availability treatment (10.5 ± 0.2 days) and slowest in the 32.2^o^C and low food availability treatment (15.6 ± 0.5 days; i.e. 50% faster). Fish from the lower feeding regimens had a lower body condition and decreased survivorship to metamorphosis. Routine oxygen consumption rates were highest for fish raised at 32.2°C and fed every third day (162 ± 107 mg O_2_  kg^−1^ h^−1^) and lowest for fish raised at 29.2°C and fed daily (122 ± 101 mg O_2_ kg^−1^ h^−1^; i.e. 35% lower). The elevated routine oxygen consumption rate, and therefore greater energy use at higher temperatures, may leave less energy available for growth and development, resulting in the longer time to metamorphosis. Overall, these results suggest that larval fishes will be severely impacted by climate-change scenarios that predict both elevated temperatures and reduced food supply.

## Abstract (precis)

We tested the impacts of temperature and variable food availability on the development and metabolic rate of the larvae of a coral reef damselfish, Amphiprion percula. Our results suggest that larval fishes will be severely impacted, both independently and synergistically, by climate-change related elevated temperatures and reductions in food supply.

## Introduction

Climate-change models predict that tropical sea surface temperatures will increase by up to 3°C this century (Ganachaud *et al.*, 2011; Lough, 2007; Meehl *et al.*, 2007). Although global warming is projected to occur more slowly in tropical than in temperate regions (Meehl *et al.*, 2007), tropical ectothermic species may be especially vulnerable to rising temperatures because many have a narrower thermal tolerance range than equivalent temperate species (Pörtner and Farrell, 2008; Sunday *et al.*, 2011) and tend to live closer to their thermal optimum; therefore, even relatively small increases in temperature could lead to declines in individual performance (Stillman, 2003; Tewksbury *et al.*, 2008). Furthermore, many biotic effects of warming are mediated through metabolic rate, which is a fundamental measure of physiological activity. In ectotherms, metabolic rate increases exponentially rather than linearly with temperature (Gillooly *et al.*, 2001; Dillon *et al.*, 2010). Consequently, an increase in metabolic rate caused by warming would require organisms to increase food intake in order to gain enough energy to cover basic functions, including growth. However, food is rarely unlimited in the natural environment; therefore, understanding the interaction between warming and food supply is important for predicting climate-change impacts on ectotherms in the tropics.

Coral reef ecosystems may be especially sensitive to ocean warming because coral bleaching and subsequent mortality is often linked to high temperatures (Glynn, 1991; Hoegh-Guldberg, 1999). Degradation of coral reef habitat has negative consequences for many coral-associated organisms, such as coral reef fish (Wilson *et al.*, 2006; Munday *et al.*, 2008a; Pratchett *et al.*, 2008). Direct physiological consequences of climate change on reef fishes will further compound these effects. Recent laboratory experiments have shown that higher temperatures, in the range predicted for the end of this century, (i.e. up to 3°C higher than current summer averages), lead to reductions in aerobic scope (Nilsson *et al.*, 2009), critical swimming speeds (Johansen and Jones, 2011), somatic growth (Munday *et al.*, 2008b), and reproductive output (Donelson *et al.*, 2010) of adult reef fishes.

Sensitivity to temperature may be magnified during early life stages (e.g. eggs, larvae), which are the key stages for mortality, dispersal, and connectivity (Houde, 1989; Wood and McDonald, 1997). Larval coral reef fishes typically exhibit increased growth rates and shorter pelagic larval durations (PLDs) with increasing temperatures within their natural temperature range (McCormick and Moloney, 1995; Wilson and Meekan, 2002; Green and Fisher, 2004; Sponaugle *et al.*, 2006; Takahashi *et al.*, 2012). However, little is known about the effects that climate-change-associated elevated temperatures, above the normal range, will have on these traits (Pankhurst and Munday, 2011). While some studies have predicted that increased temperatures will lead to increased growth, shorter PLDs, and higher survivorship (e.g. O'Connor *et al.*, 2007), others have suggested that more variable survival is likely due to a higher starvation risk associated with higher metabolic rate (Houde, 1989; Munday *et al.*, 2012), and that connectivity patterns may be altered due to shorter PLDs (Munday *et al.*, 2009).

The impact of climate change on the availability of food for larval coral reef fishes is uncertain. Elevated ocean temperatures are predicted to affect the structure of plankton communities that are a food source for larvae (Hays *et al.*, 2005; Richardson, 2008; Barton *et al.*, 2013). Changes to plankton communities will vary, but in many locations these communities may become less productive because higher temperatures favour longer, less productive planktonic food chains (McKinnon *et al.*, 2007; Morán *et al.*, 2010). Greater thermal stratification of the water column will reduce nutrient enrichment of the surface layers that are the most important for planktonic productivity (Poloczanska *et al.*, 2007; Brander, 2010; Doney *et al.*, 2012). Steinacher *et al.* (2010) reported results from four climate-change models predicting a 2–20% reduction in global marine primary production by 2100, with declines in mid to low latitudes due to reduced nutrient input into the eutrophic zone. Future changes in plankton communities will be superimposed on a resource that is inherently variable on a broad range of spatial and temporal scales (Sorokin and Sorokin, 2009). Owing to the likely effects of increasing ocean temperature on the productivity of plankton communities, and their inherent variability, it is important to understand the consequences of these changes for planktivorous organisms.

The effects of food supply on larval fishes are well known, with both faster growth and shorter PLDs being observed with increased food supply (McCormick and Molony, 1992; Green and McCormick, 1999; Meekan *et al.*, 2003; Sponaugle *et al.*, 2009). However, with regard to climate change and the potential change in plankton productivity, it is the interaction between food supply and temperature that may hold the most relevance. While the growth rate of fishes with an unlimited food supply generally increases with increasing temperature, the effects of temperature may be detrimental in food-poor environments. Higher metabolic rates with increasing temperature may lead to faster growth, but only if the availability of food is sufficient to fuel the higher metabolic demands (Munday *et al.*, 2008a). Food is rarely unlimited in the natural environment, and fish on a fixed ration may not grow as fast with increasing temperature, owing to increasing energetic demands.

In this study, we experimentally investigated the independent and interacting effects of elevated water temperature and varying food availability on the larval development and performance of the coral reef damselfish, *Amphiprion percula*. We reared larvae at three temperatures and with three food supplies in a full orthogonal design to examine effects on larval duration, larval growth, body condition, survivorship, and metabolic rate. We tested the following hypotheses: (i) elevated temperatures would decrease PLD and increase average daily growth, length at metamorphosis, body condition at metamorphosis, and survivorship to metamorphosis; (ii) decreased food supply would increase PLD and decrease daily growth, length at metamorphosis, body condition at metamorphosis, and survivorship to metamorphosis; and (iii) increased temperature would increase metabolic rate, leading to a lower performance at the same level of food availability.

## Materials and methods

### Study species and brood stock maintenance

Five breeding pairs of the coral reef damselfish (*A. percula*) were captured from reefs in the Cairns region of northern Great Barrier Reef and transported to the Marine and Aquaculture Research Facilities Unit at James Cook University, Townsville, Queensland, Australia. Pairs were maintained at 29 ± 0.5°C in 60 l outdoor aquaria and fed 0.075 g of Aquaculture Nutrition 12/20 pellets (Proaqua Pty Ltd, Coorparoo, Queensland, Australia) twice per day. Pairs were provided with half a terracotta pot for shelter and to serve as a structure for egg deposition. The pots were inspected each morning for the presence of eggs.

### Larval rearing conditions

On the afternoon when hatching was predicted (6–8 days after eggs were laid), pots containing the eggs were transferred to another 60 l aquarium (29.2°C) inside an experimental laboratory, where hatching occurred within a few hours after darkness. Larvae were reared in the 60 l aquarium for two days in a semi-closed system; kept isolated during the day to facilitate feeding, then slowly flushed with filtered seawater each morning prior to light. Each morning after flushing, green *Nannochloropsis* spp. paste (Reed Mariculture, Campbell, CA, USA) was added to the water until the bottom of the aquarium could not be seen, equating to ∼4 million cells ml^−1^ (Moorhead and Zeng, 2011). This was done to dissipate light and maintain the nutritional value of the rotifers (*Brachionus* sp.) that were fed to the larvae at a density of 10 rotifers ml^−1^ each morning for the first two days. The number of larvae surviving until the third day ranged from ∼50 to 400 among clutches of eggs.

### Experimental design

Larval *A. percula* were subjected to three feeding regimens and three temperatures in a full orthogonal design (i.e. nine treatments). Temperatures were chosen to represent present-day summer averages in the Cairns region of the Great Barrier Reef where the brood stock were collected (29.2°C) and relevant end-of-century climate change projections for this location; i.e. +1.5°C (30.7°C) and +3°C (32.2°C). Food availability was manipulated by increasing the time lag between feeds, with larvae being provided with constant food daily, every second day, or every third day.

On the third morning post-hatch (before feeding), larvae from each clutch that were visually in good condition (i.e. displaying normal swimming behaviour and balance) were gently collected in a glass beaker and arbitrarily distributed among nine to 18 (one or two vessels per treatment, depending on the number of larvae in the clutch) 3 l culture vessels made of 150 mm polyvinyl chloride pipe as described by Moorhead and Zeng (2011). Five to 10 larvae were stocked in each replicate culture vessel at each treatment level. Three culture vessels were placed in each of three to six temperature-controlled water baths (one or two for each temperature treatment). The entire protocol was repeated seven times using progeny from four adult pairs until each treatment level had a starting sample size of >100 individuals in 11 replicate culture vessels per treatment (Table 1). The temperature of the water baths and the location of the vessels within the water baths were randomly modified for each run of the experiment to negate any influence of individual vessels or location within the laboratory on results.

**Table 1:** Number of culture vessels (replicate tanks), number of larval *Amphiprion percula* stocked in each treatment level at the start of the experiment, number of vessels containing live larvae at the end of the experiment, and number of surviving larvae among vessels at the conclusion of the experiment

Temperature (°C)	Food availability	Number of vessels	Number of larvae stocked at start	Number of vessels with surviving larvae	Number of surviving larvae among vessels
29.2	L	11	104	7	20
29.2	M	11	105	6	23
29.2	H	11	105	8	51
30.7	L	11	105	8	21
30.7	M	11	105	7	37
30.7	H	11	105	9	46
32.2	L	11	105	8	28
32.2	M	11	105	8	38
32.2	H	11	105	8	36

Abbreviations: H, fed every day; L, fed every third day; and M, fed every second day.

All larval fish were fed immediately after transfer to the experimental set-up. In addition to the rotifers, their diet was enriched with newly hatched *Artemia* sp. nauplii (INVE technologies, Amphur Pakkred, Thailand) fed at a rate of 1 ml^−1^. The vessels were ‘greened’ with *Nannochloropsis* spp. as described in the previous subsection. Temperatures were increased slowly (by 0.5–1°C every 8 h, dependent on treatment) over 24 h to reach treatment levels. Feeding manipulations began the day after transfer. Water exchange of vessels was carried out each morning using filtered seawater before the lights came on to flush out uneaten prey and faeces. The photoperiod was maintained at 14 h light–10 h dark during the trials.

Larvae were carefully checked for metamorphosis by torchlight each morning before they had an opportunity to feed. Larvae were considered metamorphosed when their post-orbital stripe became fully pigmented, which always occurred between eight and 19 days post hatch. This pigmentation coincides with a shift in habitat use and a change to benthic colouration (I. M. McLeod, personal observation), which is typical of most damselfishes (McCormick *et al.*, 2002) and has been used as a diagnostic tool for metamorphosis and settlement for a congeneric species, *Amphiprion melanopus* (Green and McCormick, 1999).

### Standard length and body condition

Metamorphosed larvae were removed from culture vessels and killed using an overdose of clove oil. Larvae were then immediately transferred to a 4% phosphate-buffered formaldehyde solution, and the following measurements were taken within 48 h. Larvae were removed from the preservative, blotted dry, weighed (to the nearest 0.1 mg), and photographed in a lateral position on a 0.5 mm plastic grid. Standard length (SL) to the nearest 0.01 mm was estimated for each fish from the digital photograph using image analysis software (ImageJ version 1.45 s; National Institutes of Health, USA). Body condition (hereafter, condition factor) was calculated as the blotted weight at metamorphosis after controlling for standard length at metamorphosis using analysis of covariance (ANCOVA).

### Pelagic larval duration and average daily growth

Individual PLD was calculated as the number of days between hatching and metamorphosis. Larval growth was assumed to be linear during the larval stage, as it is for the congener *A. melanopus* (Green and Fisher, 2004). Individual growth rates were estimated according to the formula: *R*_g_ = (*L*_m_ − *L*_h_)/*T*_m_, where *R*_g_ is the rate of growth in millimetres per day, *L*_m_ is the standard length (in millimetres) at metamorphosis, *L*_h_ is the standard length at hatching (=3.79 mm), and *T*_m_ is the time (in days) from hatching to metamorphosis. Standard length at hatching was determined by sampling 10 newly hatched *A. percula* each from three clutches. These larvae were measured using the same methodology as for the metamorphosed juveniles. The mean standard length at hatching among clutches was used for the hatch length in the above calculations.

### Survivorship

Survivorship was calculated as the number of larvae placed into each vessel, minus the number of larvae that did not survive to metamorphosis.

### Larval respirometry

Intermittent-flow respirometry was used to determine routine O_2_ consumption rates (*M^·^*O_2Routine_), a standard measure of metabolic rate (Willmer *et al.*, 2005). Oxygen consumption rates were measured at 8 days post-hatch for larvae raised at the lowest (29.2°C) and highest (32.2°C) temperatures and the lowest (food available every third day) and highest (food available daily) food availability treatments. A total of 43 larvae were tested (seven to 16 per treatment). Larvae were starved for 24–26 h before trials began.

Larvae were placed individually into 4.8 ml glass vials that served as respirometry chambers. Chambers were submerged in an aquarium maintained at the same temperature at which the larvae were reared. To reduce light levels and external disturbance, a lid was placed over the opaque aquarium during the habituation period and left in place during measurements of *M^·^*O_2Routine_. Water was continuously recirculated within each respirometer using a closed circuit connected to a peristaltic pump, which ensured homogeneous O_2_ tension throughout the apparatus. The total volume of the respirometer chamber and tubing circuit was 1 ± 1 ml. A submersible pump connected to a timer was used to flush the respirometer chambers with aerated water intermittently at 5 ml min^−1^, and excess water flushed from each respirometer overflowed from a small standpipe that extended immediately above the water surface in the aquarium.

Preliminary observations indicated that larvae took 15 min in the respirometer to exhibit normal behaviour, an observation that was supported by *M^·^*O_2Routine_ measurements plateauing after this time (I. M. McLeod, unpublished data). To avoid the early period of stress, *M^·^*O_2Routine_ measurements commenced ∼15 min after the larvae entered the respirometer and continued for a total of four 15 min measurements separated by 5 min flush cycles.

The temperature-compensated O_2_ concentration of the water within each chamber was continuously recorded (1 Hz) using oxygen-sensitive REDFLASH dye on contactless spots (2 mm) adhered to the inside of each chamber and linked to a Firesting Optical Oxygen Meter (Pyro Science e. K., Aachen, Germany) via fibre-optic cables. To reduce background bacterial O_2_ consumption, seawater used for the respirometers was UV sterilized, and the system was cleaned with 70% ethanol each day, or more often if background respiration exceeded 10%. In addition, background respiration was measured before and after each trial and used to correct fish *M^·^*O_2Routine_ measurements assuming a linear change in background respiration (i.e. subtracted from the values calculated for the whole animal). At the end of the respirometry trials, larvae were killed with an overdose of clove oil, blotted dry with a paper towel, and weighed with scales accurate to 0.1 mg.

### Data analysis

Two-factor ANOVA followed by Tukey's *post hoc* comparisons of means were used to test the effects of temperature and food availability on PLD, average daily growth, standard length at metamorphosis, individual metabolic rate, and weight-adjusted metabolic rate. The assumptions of normality and homogeneous variances for each performance variable were tested using Levene's test and graphically analysed using residual and Q–Q plots. A natural log transformation was required for the PLD data and a square root transformation was required for individual metabolic rate data to conform to the assumption of homogeneity of variance.

Condition factor at metamorphosis was compared among treatments using a two-factor ANCOVA. Temperature and food availability were the independent variables, weight at metamorphosis was the dependent variable, and standard length at metamorphosis was the covariate. The adjusted weight at metamorphosis from this analysis, which controlled for standard length at metamorphosis, was used as our measure of body condition (condition factor). There was a linear relationship between weight and standard length for each treatment, as assessed by visual assessment of a scatterplot. Standardized residuals for the treatments and for the overall model were normally distributed, as assessed by Shapiro–Wilks test. There was homoscedasticity and homogeneity of variances, as assessed by visual inspection of a scatterplot, and Levene's test of homogeneity of variance, respectively. *Post hoc* analysis for the ANCOVA was performed with a Bonferroni adjustment.

Individual fish within vessels were pooled across vessels for the analysis. A nested ANOVA design was not appropriate, because some vessels had only one fish surviving to metamorphosis. Using the mean value for each vessel produced near-identical results in performance variables (revealed through ANOVA and ANCOVA). The exception was average daily growth, where there was no significant interaction between the temperature and food availability treatments using the mean vessel values, but there was a significant interaction when individuals were pooled among vessels. This was because of low statistical power due to the limited number of vessels with surviving larvae per treatment (six to nine among trials; Table 1). Partial eta-squared (η^2^) was calculated as part of the ANOVA and ANCOVA analyses, to provide a standardized measure of the relative effects of treatments on performance variables.

There were significant differences in performance variables among clutches, but consistent trends in effects of temperature and food availability (revealed through careful analysis of stem-and-leaf plots and histograms). Given that clutch effects were not the focus of the present study, individuals were pooled across clutches for the analysis. Logistic regression was used to ascertain the effects of temperature and food availability on the likelihood that individual larvae survived to metamorphosis. Individual larvae were also pooled across vessels for this analysis.

Larval respirometry data for a total of 43 larvae were analysed using LabChart version 6.1.3, (AD Instruments, Colorado Springs, CO, USA). The *M^·^*O_2Routine_ (in milligrams per kilogram per hour) was calculated from the average of the final three slopes of O_2_ concentration vs. time, minus the background O_2_ consumption. The *Q*_10_ temperature coefficient was calculated using the following formula: *Q*_10_ = (*R*2/*R*1)^10/(*T*2 −*T*1)^, where *T*1 and *T*2 are the temperatures over which the change was recorded, *R*1 is the value of the measured variable at *T*1, and *R*2 is the value of the variable at *T*2. All statistical analyses were conducted using the statistical package SPSS Statistics version 20 (IBM™ SPSS™ Inc. 2011).

## Results

### Pelagic larval duration and growth

**Figure 1:**
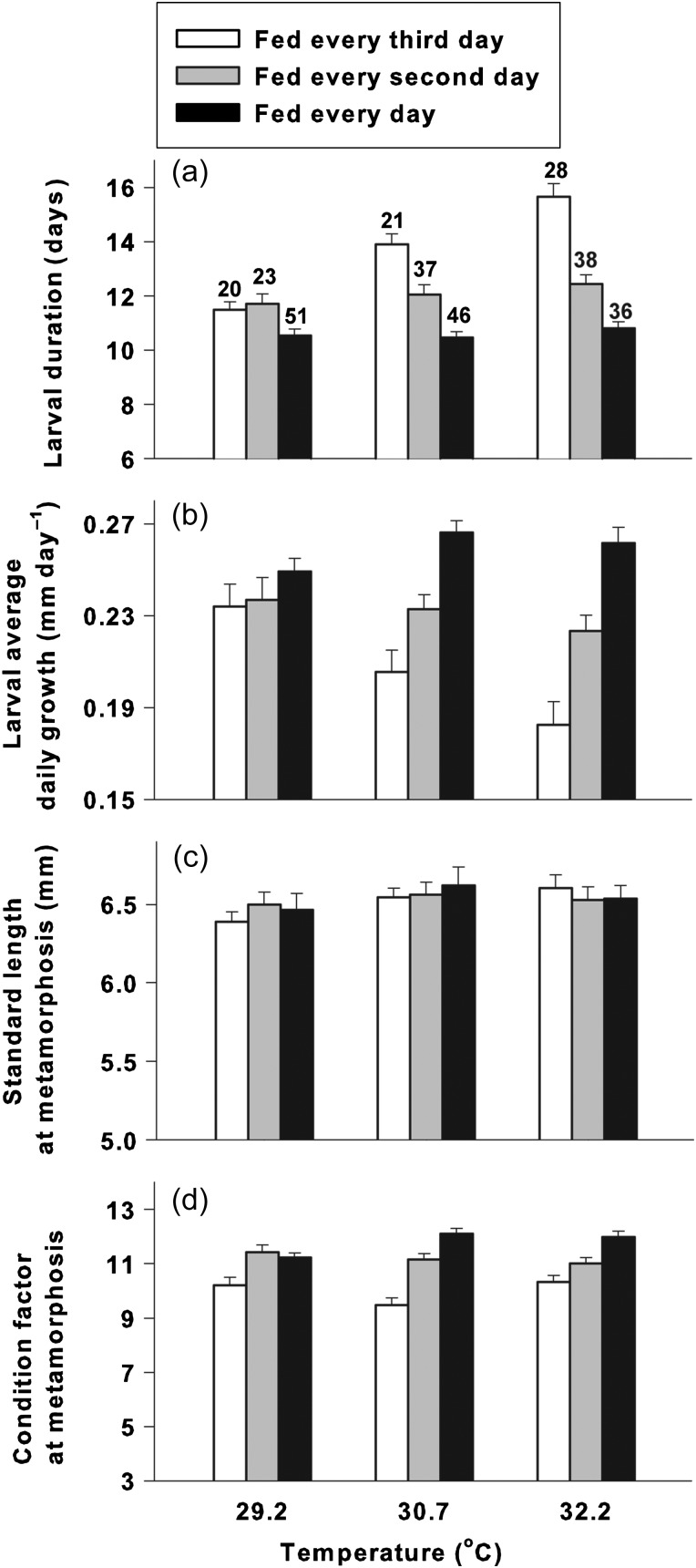
*Mean pelagic larval duration (a), larval average daily growth (b), standard length at metamorphosis (c), and body condition at metamorphosis (expressed as weight in milligrams adjusted for standard length; d) for Amphiprion percula* raised at 29.2, 30.7, and 32.2°C on a low (open bars), medium (shaded bars) or high level of food availability (filled bars). Error bars denote ± SEM. The numbers above bars indicate numbers of larvae included in the analysis**Q10**.

The average PLD of *A. percula* ranged from 10.5 ± 0.2 days (mean ± SEM) in the 30.7°C and high food availability treatment to 15.6 ± 0.5 days in the 32.2°C and low food availability treatment (i.e. a 50% increase; Fig. 1a). There was a significant interaction (*F*_4,291_ = 6.4, *P* < 0.001, partial η^2^ = 0.081) between temperature and food availability on PLD (Table 2). Access to food had a stronger effect (partial η^2^ = 0.286) than temperature (partial η^2^ = 0.093). The significant interaction was due to a much greater effect of food availability on PLD at elevated temperatures compared with the baseline temperature (29.2°C). *Post hoc* tests showed that at 29.2°C the individual PLDs were significantly longer in the medium (fed every second day) than in the high (fed every day) food availability treatment. At 30.7°C, PLDs were significantly longer for the medium and low food availability treatments compared with the high food availability treatment, and PLDs were significantly longer for the low compared with the medium food availability treatment.

**Table 2:** ANOVA and ANCOVA table for pelagic larval duration, larval average daily growth, standard length at metamorphosis, condition at metamorphosis (ANCOVA), and routine metabolism (*M^·^*O_2Routine_; individual and weight adjusted) for larval *A. percula* reared in nine combinations of water temperature and food availability

Source	d.f.	Mean square	*F*	*P* value	Effect size (partial η^2^)
Pelagic larval duration					
Temperature	2	0.366	15	<0.0001*	0.093
Food availability	2	1.42	58.3	<0.0001*	0.286
Temperature × food availability	4	0.156	6.39	<0.0001*	0.081
Error	291	0.024			
Average daily growth					
Temperature	2	0.0000748	4.198	0.016*	0.028
Food availability	2	0.001	35.132	<0.0001*	0.194
Temperature × food availability	4	0.0000821	4.609	0.001*	0.06
Error	291	0.0000178			
Standard length at metamorphosis					
Temperature	2	0.381	1.782	0.17	0.012
Food availability	2	0.018	0.084	0.919	0.001
Temperature × food availability	4	0.092	0.431	0.786	0.006
Error	291	0.214			
Condition factor at metamorphosis (ANCOVA)					
Length (covariate)	1	0.001	458.26	<0.0001	0.612
Temperature	2	1.15	0.687	0.504	0.005
Food availability	2	69.8	41.944	<0.0001*	0.224
Temperature × food availability	4	7.346	4.41	0.002*	0.057
Error	290	1.67			
Individual *M·*O_2Routine_					
Temperature	1	0	19.95	<0.0001*	0.216
Food availability	1	0	10.7	0.002*	0.338
Temperature × food availability	1	0.0000255	2.56	0.117	0.062
Error	39	0.00001			
Individual *M·*O_2Routine_ (weight adjusted)					
Temperature	1	306	6.48	0.015*	0.142
Food availability	1	1.82	0.039	0.845	0.001
Temperature × food availability	1	1.94	0.041	0.84	0.001
Error	39	47.2	Error	39	47.2

There was also a significant interaction (*F*_4,291_ = 4.6, *P* = 0.01, partial η^2^ = 0.060) between temperature and food availability on the average growth rate between hatching and metamorphosis (Table 2 and Fig. 1b). Again, the food availability had a stronger effect (partial η^2^ = 0.194) than temperature (partial η^2^ = 0.0280). The interaction was due to a much greater effect of food availability on average daily growth at elevated temperatures compared with the baseline temperature (29.2°C). Specifically, temperature had no effect on growth for the high or medium food availability treatments, but for the low food availability treatment, growth was significantly lower at 32.2 than at 29.2°C. Growth ranged from 0.27 ± 0.0052 mm day^−1^ in the 30.7°C and high food supply treatment to 0.018 ± 0.01 mm day^−1^ in the 32.2°C and low food availability treatment (i.e. a 31% decrease; Fig. 1b).

### Standard length at metamorphosis

*Amphiprion percula* metamorphosed at a standard length of 6.5 ± 0.5 mm across all treatments (Fig. 1c). There was no significant effect of temperature (*F*_2,291_ = 1.8, *P* = 0.17, partial η^2^ = 0.012) or access to food (*F*_2,291_ = 0.09, *P* = 0.92, partial η^2^ = 0.001) on SL at metamorphosis, and no interaction between temperature or food availability on SL at metamorphosis (*F*_4,291_ = 0.43, *P* = 0.79, partial η^2^ = 0.006; Table 2).

### Body condition (condition factor) at metamorphosis

After adjusting for standard length at metamorphosis (through an ANCOVA), there was a significant interaction (*F*_4,290_ = 4.41, *P* = 0.02, partial η^2^ = 0.057) between temperature and food availability on the average blotted weight at metamorphosis (condition factor; Table 2 and Fig. 1d). The significant interaction was due to a greater effect of food availability for the 30.7°C treatments than at 29.2°C. Mean condition factor ranged from 11.2 at 30.7°C and with high food availability to 9.47 with the low food availability at that temperature. Food availability (partial η^2^ = 0.224) had a stronger effect than temperature (partial η^2^ = 0.005). Condition factor was always lower in the low food availability treatments than in the high or medium food availability treatments.

### Survival to metamorphosis

Among treatments, 31.8% of larvae survived to metamorphosis, ranging from 19.0% in the 29.2°C, low food availability treatment to 49.0% in the 29.2°C, high food availability treatment (Table 1). The logistic regression model (*x*^2^(2) = 16, *P* < 0.001) explained 49.7% of the variance in survival to metamorphosis and correctly classified 62.7% of cases. Food availability but not temperature was a statistically significant predictor of survival to metamorphosis. Larvae in the high food availability treatments (42.4% survival) were 1.4 times more likely to survive than those in the medium food availability treatments (31.1%), and 1.9 times more likely to survive to metamorphosis than those in the low food availability treatments (21.9%).

### Larval respirometry

**Figure 2:**
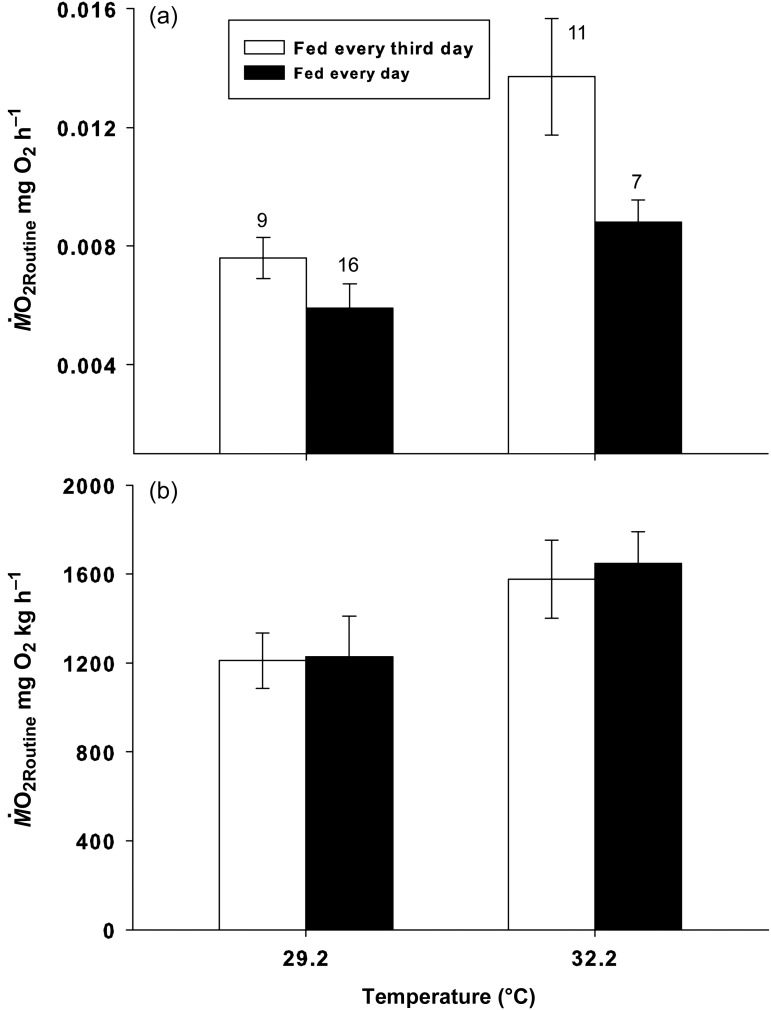
Mean routine individual oxygen consumption rates (*M^·^*O_2Routine_; expressed as milligrams of O_2_ per hour; a) and weight-adjusted individual oxygen consumption rates (*M^·^*O_2Routine_; expressed as milligrams of O_2_ per kilogram per hour; b) for 8-day-old *A. percula* raised at 29.2 and 32.2°C on a low (open bars) or high level of food availability (filled bars). Error bars denote ± SEM. The numbers above bars indicate numbers of larvae included in the analysis.

Both temperature (*F*_1,39_ = 20, *P * < 0.0001, partial η^2^ = 0.338) and food availability (*F*_1,39_ = 10.7, *P* = 0.002, partial η^2^ = 0.216) significantly affected individual larval O_2_ consumption. However, there was no interaction between temperature and food availability (*F*_1,39_ = 2.56, *P* = 0.117, partial η^2^ = 0.062). Mean O_2_ consumption ranged from 0.0059 ± 0.00082 mg O_2_ h^−1^ for larvae at 29.2°C and fed every third day to 0.013 ± 0.0059 mg O_2_ h^−1^ for larvae at 32.2°C and fed every day (i.e. a 120% increase; Fig. 2a). However, fish in the high food availability treatments were heavier on day 8 when they were tested. When *M^·^*O_2Routine_ was corrected for the individual weights of the larval fish, temperature (*F*_1,39_ = 6.5, *P* = 0.015, partial η^2^ = 0.142; Fig. 2b) but not food supply (partial η^2^ = 0.001) significantly affected *M^·^*O_2Routine_, with no significant interaction. Mean *M^·^*O_2Routine_ was 122 ± 101 mg O_2_ kg^−1^ h^−1^ at 29.2°C and 162 ± 107 mg O_2_ kg^−1^ h^−1^ at 32.2°C (i.e. a 33% increase). The *Q*_10_ coefficient for *M^·^*O_2Routine_ calculated over this 3°C increase in temperature was 2.59.

## Discussion

Our results suggest that climate-induced increases in ocean temperature and variation in planktonic food supply may impact the development and metabolism of larval reef fishes. Overall, larval *A. percula* grew more slowly and took longer to metamorphose and settle at higher temperatures and with reduced access to food, with a highly significant interaction between these factors. However, neither temperature nor access to food affected the length at metamorphosis. Fish from the lower access to food treatments had a lower condition factor and decreased survivorship. Temperature affected routine oxygen consumption rates (*M^·^*O_2Routine_), which were significantly higher at 32.2°C than at 29.2°C. The differences in individual *M^·^*O_2Routine_ between the high and low food availability treatments were explained by the lower weight of the larvae fed every third day. When the metabolic rate was corrected for individual weight, there was no influence of food availability on *M^·^*O_2Routine_.

The direct effects of temperature included an increase in PLD, a lower growth rate, and an increase in metabolic rates at higher temperatures. Previous research investigating the effects of temperature on larval coral reef fish growth and PLD showed that higher temperatures lead to increased growth and shorter PLDs (e.g. McCormick and Moloney, 1995; Green and Fisher, 2004; Sponaugle *et al.*, 2006; Takahashi *et al.*, 2012). Furthermore, in a recent review, Munday *et al.* (2009) postulated that the limited evidence available suggests that a 3°C increase in sea surface temperatures would reduce the PLD of larval reef fishes by 12–25%. However, this estimate was made by extrapolating results from experiments and field studies looking at temperatures within their natural range, because there were few data available regarding the effects of elevated temperatures on these traits. Importantly, our results show that developmental rates and PLDs may not continue a linear relationship with temperature beyond the range of temperatures typically experienced by the population. Consequently, extrapolations based on present-day variation may be inappropriate for projecting the consequences of future temperature increases on the early life history traits of tropical fishes.

Food availability affected survivorship to metamorphosis, with reduced survivorship in the lower food availability treatments. *Amphiprion percula* are capable of feeding immediately upon hatching, and feeding treatments were not commenced until the fourth day post-hatch. Hjort (1914) first emphasized the importance of food limitation at the time of first feeding for fish larvae. If food was reduced over the first three days, overall survivorship may have been reduced further, suggesting that our findings, although substantial and significant, may still be conservative. In nature, mortality is extreme in the larval phase, and so small changes in mortality rates could have important ramifications for recruitment to adult populations (Jones, 1991; Leis, 1991).

Access to food had a major impact on growth rates and PLD, with longer PLDs and slower average growth rates in the lower food availability treatments. Food availability also had a profound effect on condition factor at metamorphosis, with lower food availability treatments resulting in lower weight for a given body length. These results are consistent with previous experiments that tested the effects of reduced food for larval coral reef fish (e.g. McCormick and Molony, 1992; Green and McCormick, 1999). Lowered condition factor and growth associated with suboptimal feeding in the larval environment is likely to affect the likelihood of survival well after the fish have metamorphosed and settled (Hoey and McCormick, 2004; Gagliano *et al.*, 2007; McCormick and Gagliano, 2009). Poor feeding history has a lasting effect on metabolism, and this may lead to behavioural trade-offs associated with the balance between feeding and predator vigilance (Metcalfe and Monaghan, 2001). Fishes that have had poor growth histories sometimes compensate by over-performing in growth once feeding conditions improve, and this over-performance in growth can be at the cost of behaviours that directly affect survival (Pechenik, 1990; Gagliano and McCormick, 2007).

The interacting effects of elevated temperature and reduced food availability resulted in much longer PLDs and slower growth rates, suggesting that the harmful effects of ocean warming are likely to be most severe if accompanied by a declining planktonic food supply. The increase in PLD would be likely to lead to important ecological consequences, because the larval life stage has the highest risk of mortality through predation (Houde, 1987; Bailey and Houde, 1989). A longer PLD increases the length of time that larval fish are exposed to the high-risk pelagic environment, thus indirectly reducing the probability of survival. This contrasts with predictions of increased larval survival by some analyses that have primarily synthesized PLD data for many species within their present-day temperature range (e.g. O'Connor *et al.*, 2007). Predictions of increased larval survival may be inaccurate, especially for tropical species, if they do not account for the shape of the thermal reaction norm in PLD above the range of natural variation and incorporate the possible effects of reduced food supply (Steinacher *et al.*, 2010). For those larvae that survive longer PLDs, this might have some implications for the spatial scale of larval dispersal, and thus, the scale of connectivity for some populations, which could have flow-on effects for population dynamics and sustainability (Munday *et al.*, 2009).

It is difficult to estimate the relative importance of temperature and food supply for larval performance in the wild using the results of this study, because very little is known about the temporal variability in food supply and larval feeding in the wild. Owing to this lack of information and logistical constraints, the food treatments used in the present study were chosen to represent high, medium, and low access to food for comparative purposes, and may not be indicative of natural food supply. Nevertheless, they enabled us to examine experimentally the relationship between metabolism and food availability, and how performance variables are affected. In our experiments, the difference in effect sizes show that food supply was more important than temperature for PLD, larval growth, and condition factor. These results contrast with those of Meekan *et al.* (2003), who showed that over two consecutive summers, water temperature explained 30% and zooplankton abundance only 3.5–4.1% of the variance in growth for a tropical damsel fish, *Pomacentrus coelestis*.

In our study, metabolic rate increased with increased temperature, a result consistent with findings from previous studies (reviewed by Houde, 1989; Rombough, 1997; Peck *et al.*, 2012). In a recent review, Peck *et al.* (2012) found that a 10°C increase in temperature was accompanied by a 1.2- to 4.3-fold increase in larval *M^·^*O_2Routine_ (*Q*_10_ = 1.2–4.3) across 15 families of marine fish. In the present study, the *Q*_10_ was ∼2.6, similar to the average *Q*_10_ (2.31) found by Peck *et al.* (2012). Elevated *M^·^*O_2Routine_ and therefore energy use at higher temperatures may leave less energy available for growth, resulting in the longer time to metamorphosis, especially when food supplies are low. In our laboratory experiment, the tanks were static, with no flow during the day, and with high concentrations of nutritious prey, so overall energy use is likely to be lower than in nature. Consequently, the effects of reduced access to food may be more severe in the natural environment than the results of this experiment indicate, because larvae in nature must swim against ocean currents and are likely to consume prey of lesser nutritional value.

Larvae settled at a remarkably consistent length across treatments, indicating that reaching a precise size was important for metamorphosis in *A. percula*. Fish in the lower access to food treatments also had a lower condition factor, indicating that it is taking longer for the fish in the lower food availability treatments to become competent to settle, rather than delaying metamorphosis as some other coral reef will do when denied access to the preferred settlement structure (McCormick, 1999). Across marine fish species, there is more variation in age than size at metamorphosis (Chambers and Leggett, 1987). However, our results contrast with results for a congener, *A. melanopus*, in which length at settlement was significantly longer at lower temperatures (Green and Fisher, 2004) and shorter in lower food availability treatments (Green and McCormick, 1999). A larger size at settlement may offer some survival advantages (Sogard, 1997; Perez and Munch, 2010). Perhaps the disadvantage of settling at a smaller size outweighs the potential reduction in survivorship that would result from an extended PLD.

### Conclusions

This study highlights the potential interacting effects that higher ocean temperatures and reductions in food supply will have on larval coral reef fishes. The *M^·^*O_2Routine_ was higher at higher temperatures, indicating a potential mechanism for the differences in growth and development in relationship to food supply. Indeed, the greater effect of temperature in the lowest food availability conditions could be because there is insufficient food to meet the increased metabolic costs. Overall, our results indicate that despite previous predictions of a positive influence of ocean warming on larval development and survival, variable food supply alters how temperature affects growth and development. These observations demonstrate that increased temperature and reduced food supply will affect the life history and demography of larval fish, possibly affecting recruitment processes and population dynamics. Further work is required to determine whether these results are general for a range of other fish species. The potential for coral reef fish populations to adapt to climate-change-associated elevated temperatures and variable access to food during the larval phase is unknown, but is a critical area of future research (Munday *et al.*, 2009, 2012). Future studies could use quantitative genetic breeding designs (Lynch and Walsh, 1998) to test the heritability of individual variation in the response of larval fish to elevated temperature and reduced food supply. Such experiments are logistically challenging, but ultimately essential for predicting the potential for populations to adapt to rapid climate change. This work is urgent, given the potential susceptibility of fish larvae to almost every predicted change facing the oceanic environment.
